# A Latent Markov Modelling Approach to the Evaluation of Circulating Cathodic Antigen Strips for Schistosomiasis Diagnosis Pre- and Post-Praziquantel Treatment in Uganda

**DOI:** 10.1371/journal.pcbi.1003402

**Published:** 2013-12-19

**Authors:** Artemis Koukounari, Christl A. Donnelly, Irini Moustaki, Edridah M. Tukahebwa, Narcis B. Kabatereine, Shona Wilson, Joanne P. Webster, André M. Deelder, Birgitte J. Vennervald, Govert J. van Dam

**Affiliations:** 1 MRC Centre for Outbreak Analysis and Modelling, Department of Infectious Disease Epidemiology, Imperial College London, London, United Kingdom; 2 Department of Statistics, London School of Economics and Political Science, London, United Kingdom; 3 Schistosomiasis Control Initiative at Vector Control Division – Ministry of Health, Kampala, Uganda; 4 Department of Pathology, University of Cambridge, Cambridge, United Kingdom; 5 Department of Infectious Disease Epidemiology, Imperial College London, London, United Kingdom; 6 Department of Parasitology, Leiden University Medical Center, Leiden, The Netherlands; 7 Section for Parasitology and Aquatic Diseases, University of Copenhagen, Copenhagen, Denmark; University of New South Wales, Australia

## Abstract

Regular treatment with praziquantel (PZQ) is the strategy for human schistosomiasis control aiming to prevent morbidity in later life. With the recent resolution on schistosomiasis elimination by the 65th World Health Assembly, appropriate diagnostic tools to inform interventions are keys to their success. We present a discrete Markov chains modelling framework that deals with the longitudinal study design and the measurement error in the diagnostic methods under study. A longitudinal detailed dataset from Uganda, in which one or two doses of PZQ treatment were provided, was analyzed through Latent Markov Models (LMMs). The aim was to evaluate the diagnostic accuracy of Circulating Cathodic Antigen (CCA) and of double Kato-Katz (KK) faecal slides over three consecutive days for *Schistosoma mansoni* infection simultaneously by age group at baseline and at two follow-up times post treatment. Diagnostic test sensitivities and specificities and the true underlying infection prevalence over time as well as the probabilities of transitions between infected and uninfected states are provided. The estimated transition probability matrices provide parsimonious yet important insights into the re-infection and cure rates in the two age groups. We show that the CCA diagnostic performance remained constant after PZQ treatment and that this test was overall more sensitive but less specific than single-day double KK for the diagnosis of *S. mansoni* infection. The probability of clearing infection from baseline to 9 weeks was higher among those who received two PZQ doses compared to one PZQ dose for both age groups, with much higher re-infection rates among children compared to adolescents and adults. We recommend LMMs as a useful methodology for monitoring and evaluation and treatment decision research as well as CCA for mapping surveys of *S. mansoni* infection, although additional diagnostic tools should be incorporated in schistosomiasis elimination programs.

## Author Summary

Schistosomiasis remains one of the most prevalent parasitic diseases in developing countries, with *Schistosoma mansoni* being the most widespread of the human-infecting schistosomes. For the routine surveillance of human *S. mansoni* infection more “field-applicable,” sensitive, and cost-effective diagnostics that replicate faecal samples over several consecutive days [the Kato-Katz (KK) method], are needed. We propose a statistical modelling framework in order to evaluate the diagnostic performance of the urine strip test for Circulating Cathodic Antigen (CCA) and single-day double KK measurements over three consecutive days for the diagnosis of *S. mansoni* infection in two different age groups from Uganda pre- and post- praziquantel (PZQ) treatment. We demonstrate that CCA is an appropriate tool for mapping surveys of *S. mansoni* infection. Our findings should allow for evaluation of the risk of potential misinterpretation with regards to diagnosis of *S. mansoni* infection through CCA or KK in this endemic setting pre- and post- PZQ treatment as the numbers and infection intensities are brought down, bridging existing important gaps in schistosomiasis diagnostics research. More generally, the proposed statistical analysis can reveal important biological insights from other diseases without gold standard diagnostic tools whenever longitudinal data are available.

## Introduction

Schistosomiasis is a debilitating parasitic disease in tropical and sub-tropical areas which has recently attracted increased focus and funding for control through large scale mass drug administration (MDA) of praziquantel (PZQ) [1]. However the ability of current control initiatives to operate cost effectively is reduced by technical limitations of currently available schistosomiasis diagnostics [2], [3]. With the recent resolution on schistosomiasis elimination by the 65th World Health Assembly, schistosomiasis diagnostics research for population-based assessment is critical with careful consideration given to how those tools might be used within disease elimination programmes [4]–[6].

At present the World Health Organization (WHO) recommends the Kato-Katz (KK) method as the standard tool for the qualitative and quantitative diagnosis of *Schistosoma mansoni* infection because of its assumed high specificity, relative simplicity in field conditions and attractive price. WHO also recommends MDA with PZQ to be delivered to community populations defined where KK surveys show an estimated prevalence of over 50% in school-aged children and to be delivered to children aged 6–16 years where the estimated prevalence is between 10% and 50% in this age group. However, it is well known that KK method from single stool samples, particularly at low infection endemicities and following PZQ MDA, can underestimate *Schistosoma* infection prevalence (and intensities) and thus overestimate cure rates [7]–[11], whilst even multiple slides over multiple days of stool sampling can influence specificity and overestimate prevalence [10]–[15]. The necessity for more “field-applicable”, sensitive and cost-effective diagnostics than the KK method, at least for the routine surveillance of human *S. mansoni* infection such as that inherent within mapping of at-risk populations has also been recently highlighted [16], [17]. Even more worrying, in some endemic regions microscopic stool samples examination is considered too logistically difficult in terms of personnel available for routine surveillance and therefore *S. mansoni* infection remains undetected and untreated in control programmes [18]. A promising diagnostic option is a urine strip test for Circulating Cathodic Antigen (CCA) which is a genus-specific glycan regurgitated by adult schistosome worms into the blood stream [19]. A number of cross-sectional studies evaluating CCA accuracy pre-treatment have stressed the need for further research assessing the potential role of this diagnostic assay at different stages of schistosomiasis control programs [15], [17], [20].

In this study, we assessed for the first time the diagnostic accuracy of CCA and of double KK faecal slides over three consecutive days for *S. mansoni* infection by age group at baseline and at two follow-up times post treatment which should be viewed as a proxy for low transmission areas. Because no true gold standard diagnoses are available, we developed and fitted latent Markov models (LMMs) to estimate diagnostic test sensitivities and specificities, the true underlying infection prevalence and the probabilities of transitions between infected and uninfected states [21]. LMMs - which are sometimes also referred to as latent transition models or regime-switching models – are used to analyze discrete-time longitudinal data where respondent observations contain measurement error. This approach defines the true states as categories (latent classes) of a dynamic latent (unobservable) variable within a statistical model. The Markov assumption is reflected in the model via transition probabilities which allow for correlation between a respondent's true state at times *t*−1 and *t* [22].

We analyzed a detailed dataset from a longitudinal cohort living along the shorelines of Lake Victoria in Uganda who received one or two doses of PZQ treatment at baseline. We demonstrated how the use of LMMs allows estimation of the ‘true’ prevalence of *S. mansoni* infection over time and the quantification of the additional benefit of a second PZQ dose in reducing re-infection levels by age group.

## Materials and Methods

### Ethics Statement

Ethical clearance was obtained from the Uganda National Council of Science and Technology and the study was also presented to the Danish National Committee on Biomedical Research Ethics in Denmark (Reference Number: UNCST: HS 59). Informed consent was obtained from individual adult participants but for children the parents or guardians consented on their behalf. Thereafter, each individual signed a consent form before any activity started. All information obtained from participants was kept confidential. Because some of the participants might have potentially received PZQ treatment recently through MDA, field survey assistants asked each participant detailed questions about previous treatment in order to exclude such individuals ever treated through MDA, although no such pre-treated individuals were identified in the current study.

### Study Design and Population

We conducted our study in Musoli village, Mayuge district at baseline and nine weeks after treatment during 2005. Participants of this study were randomly allocated to one or two PZQ (Shin Poong Pharmaceuticals, Seoul Republic of Korea) doses at baseline (40 mg PZQ per kg body weight; double treatment group: two times PZQ 40 mg per kg body weight administered two weeks apart). The field survey assistants who delivered these treatments were not aware of the infection status of any participants at any time. First follow-up data collection was performed at nine weeks , and hence at a time aimed to assess cure rates where the risk of any eggs detected occurring as a consequence of reinfection was minimal. Second follow-up data collection was performed two years later during 2007. With regards to treatment after two years, this is part of the National programme to treat everyone living in an endemic area and thus a second MDA was offered after two years. The study location was selected as this is an area of Uganda known for perennial *S. mansoni* transmission, situated on the shore of Lake Victoria [23] where the community population is not targeted by the National Control Program [24], [25]. The community consists primarily of fishermen and their dependants with the lake being the only source of fresh water for them. Furthermore, infrequent use and/or availability of latrines leads to contamination of the lake especially near the shoreline, where there is underwater vegetation suitable for the aquatic intermediate host snails to thrive. In addition, only individuals >6 years of age were enrolled as very often this is the age from which schistosome-induced morbidity, in general, becomes evident.

### Population Census

Before any data collection took place, trained and experienced demographers conducted in 2005 a census of the village population. During this process the inhabitants in each household registered their relation to the head of the household, year of birth, gender, occupation, duration of residency in the village and tribal membership.

### Sample Size

A stratified random sample for age and sex was then selected from the census data. Sample size calculations included detection of a significant difference of cure rates between the two treatment groups with reference to cure rates obtained along Lake Albert. A significance level of α = 0.05, a power of 90% with a drop-out rate of 40% over the two years of studies contributed to the calculation of 552 individuals but at baseline we managed to recruit 446 with full parasitological and CCA in urine data as described elsewhere [23], [26]. A further six cases were then eliminated from the analysis presented in this study due to missing data in number of treatments and their age.

### Parasitological and Immunological Diagnosis

Stool samples were collected on three consecutive days from each member of the cohort and examined for the presence of *S. mansoni* ova. Two duplicate slides of each stool sample were examined using the KK technique at each day. Each slide was read by two trained microscopists and any discrepancies resolved before results were recorded as eggs per gram (EPG) faeces. The results for all six slides were combined for the descriptive results presented in Table 1 as this is considered the best KK diagnostic performance scenario [13] while results of two slides of each single day are combined and incorporated in the statistical models' derived results (Tables 2A–3B) because in many studies only a single stool sample is analysed [25], [27]–[29].

**Table 1:** 2×2 descriptive tables for *S. mansoni* infection as determined by CCA and microscopy over time; sensitivity and specificity of CCA were estimated assuming that the combination of 6 KK measurements over 3 days is 100% sensitive and 100% specific.

A: for children (age 7–16)						
	Baseline		9 weeks		2 years	
	KK (6 measurements over 3 days)					
CCA	−	+	−	+	−	+
**−**	2	17	39	26	4	7
**+**	2	148	17	66	7	101
***Empirical CCA diagnostic performance (%) assuming KK (6 measurements over 3 days) as “the gold standard”***						
***Sensitivity*** a	89.7 (84.0 to 93.9)^c^		71.7 (61.4 to 80.6)		93.5 (87.1 to 97.4)	
***Specificity*** b	50.0 (6.8 to 93.2)		69.6 (55.9 to 81.2)		36.4 (10.9 to 69.2)	

B: for adolescents and adults (age 17–76)						
	Baseline		9 weeks		2 years	
	KK (6 measurements over 3 days)					
CCA	−	+	−	+	−	+
**−**	37	41	124	24	54	29
**+**	7	189	32	43	30	80
***Empirical CCA diagnostic performance (%) assuming KK (6 measurements over 3 days) as “the gold standard”***						
***Sensitivity*** a	82.2 (76.6 to 86.9)		64.2 (51.5 to 75.5)		73.4 (64.1 to 81.4)	
***Specificity*** b	84.1 (69.9 to 93.4)		79.5 (72.3 to 85.5)		64.3 (53.1 to 74.5)	

a This is derived by taking the ratio of “true positives” over the sum of “true positives” and “false negatives”. For instance for children at baseline number of “true positives” is 148 and number of “false negatives” is 17.

b This is derived by taking the ratio of “true negatives” over the sum of “true negatives” and “false positives”. For instance for children at baseline number of “true negatives” is 2 and number of “false positives” is 2.

c 95% Exact binomial confidence intervals are provided in brackets.

General note: Complete case data at each time point are displayed and contributed to the calculations of empirical sensitivities and specificities.

**Table 2:** LMM estimated sensitivities and specificities over time (with 95% confidence intervals) with no gold standard assumed.

A: for children-n = 167								
	CCA		KK (2 measurements) 1^st^ day		KK (2 measurements) 2^nd^ day		KK (2 measurements) 3^rd^ day	
	*Sensitivity*	*Specificity*	*Sensitivity*	*Specificity*	*Sensitivity*	*Specificity*	*Sensitivity*	*Specificity*
**Baseline**	92.7 (88.3 to 100.0)	54.5 (45.1 to 63.7)	99.0 (90.4 to 100.0)	71.5 (53.4 to 84.6)	93.8 (88.6 to 100.0)	64.1 (47.9 to 77.6)	96.0 (92.4 to 100.0)	70.9 (50.8 to 85.1)
**9 weeks**	92.7 (88.3 to 100.0)	54.5 (45.1 to 63.7)	82.0 (56.9 to 100.0)	87.6 (77.5 to 93.5)	94.1 (71.2 to 100.0)	90.4 (69.1 to 97.5)	72.6 (53.6 to 100.0)	80.0 (68.5 to 88.1)
**2 years**	92.7 (88.3 to 100.0)	54.5 (45.1 to 63.7)	99.0 (90.4 to 100.0)	71.5 (53.4 to 84.6)	93.8 (88.6 to 100.0)	64.1 (47.9 to 77.6)	96.0 (92.4 to 100.0)	70.9 (50.8 to 85.1)

B: for adolescents and adults-n = 273								
	CCA		KK (2 measurements) 1^st^ day		KK (2 measurements) 2^nd^ day		KK (2 measurements) 3^rd^ day	
	*Sensitivity*	*Specificity*	*Sensitivity*	*Specificity*	*Sensitivity*	*Specificity*	*Sensitivity*	*Specificity*
**Baseline**	90.1 (84.3 to 100.0)	73.6 (68.0 to 78.6)	94.0 (89.5 to 100.0)	81.5 (74.5 to 87.0)	92.7 (87.9 to 100.0)	85.8 (78.7 to 90.8)	95.4 (90.2 to 100.0)	85.9 (79.1 to 90.8)
**9 weeks**	90.1 (84.3 to 100.0)	73.6 (68.0 to 78.6)	49.7 (35.8 to 100.0)	95.6 (90.0 to 98.2)	60.0 (41.9 to 100.0)	98.7 (93.7 to 99.8)	54.1 (38.1 to 100.0)	95.7 (90.9 to 98.1)
**2 years**	90.1 (84.3 to 100.0)	73.6 (68.0 to 78.6)	94.0 (89.5 to 100.0)	81.5 (74.5 to 87.0)	92.7 (87.9 to 100.0)	85.8 (78.7 to 90.8)	95.4 (90.2 to 100.0)	85.9 (79.1 to 90.8)

The measurement invariance hypothesis for CCA was accepted and thus estimates of sensitivities and specificities for this diagnostic test remain constant over time.doi:10.1371/journal.pcbi.1003402.t002

**Table 3:** LMM estimated transition probability matrices.

A: for children-n = 167						
Treatment Interval						
Baseline-to-9 weeks						
		9 weeks				
		*1 PZQ dose*			*2 PZQ doses*	
		I−	I+		I−	I+
**Baseline**	**I−**	1.000^a^	0.000	**I−**	1.000^b^	0.000
	**I+**	0.458	0.542	**I+**	0.717	0.283

Non-Treatment Interval						
9 weeks-to-2 years						
		2 years				
		*1 PZQ dose*			*2 PZQ doses*	
		I−	I+		I−	I+
**9 weeks**	**I−**	0.437	0.563	**I−**	0.422	0.578
	**I+**	0.202	0.798	**I+**	0.000	1.000^c^

B: for adolescents and adults-n = 273						
Treatment Interval						
Baseline-to-9 weeks						
		9 weeks				
		*1 PZQ dose*			*2 PZQ doses*	
		I−	I+		I−	I+
**Baseline**	**I−**	1.000^a^	0.000	**I−**	1.000^b^	0.000
	**I+**	0.561	0.439	**I+**	0.846	0.154

Non-Treatment Interval						
9 weeks-to-2 years						
		2 years				
		*1 PZQ dose*			*2 PZQ doses*	
		I−	I+		I−	I+
**9 weeks**	**I−**	0.858	0.142	**I−**	0.836	0.164
	**I+**	0.126	0.874	**I+**	0.000	1.000^c^

a, b, c These parameters were estimated close to 1 or 0 and so in order to avoid numerical instability in the estimation algorithm, MPLUS fixed these automatically to 1 or 0 respectively. Such values should be treated with caution due to computational limitations in these categories during the model estimation.

General note: The displayed results are derived by exploiting in the final LMM the interrelationships between CCA and double Kato-Katz (KK) faecal slides over 3 consecutive days as well as not assuming a gold standard.

Single urine samples were kept cold after collection and after return to the laboratory at the end of the day aliquoted and stored frozen. At baseline and nine weeks, CCA urine samples were kept in a freezer in Uganda for nine months before they were transferred frozen to Leiden. At the two year follow-up CCA urine samples were delivered to Leiden within four weeks of collection. In Uganda, all CCA urine samples were kept at −20 Celsius degrees. The freezer in which urine was stored was −18 to −20 degrees C. Results should not be affected as the antigen is very stable and its detection is not influenced by periods of being frozen, freeze thaw cycles, or even storage for weeks at room temperature. They were kept frozen during transport to the Department of Parasitology, in Leiden, The Netherlands, where the CCA urine assays were performed as previously described [19], [30]. Briefly, for the laboratory-based test, 25 µL of completely thawed and vortexed urine was added to a tube containing dried carbon conjugated antibody, along with 75 µL of buffer and mixed well. Test strips were added, and allowed to develop for 40 minutes. Strips were removed, allowed to dry, and read against a set of five Quality Control (QC) standards of 0, 10, 100, 1000 and 10 000 ng of semi-purified worm antigen (containing CCA) per ml negative urine. A score of 0 indicated a negative result; the 0.5 stands for trace, while 1, 2, and 3, indicated that the intensity of the test line was similar to that of the respective 100, 1000, and 10 000 ng/ml spiked QC samples. Strips were scored in a blinded fashion by at least two individuals and in case of discrepancies a third person was consulted to conclude on the score. The classification of the trace result is decided later (see ‘Model selection’ section) based on the use of specific latent variable models. All other positive results (scores 1, 2 and 3) were merged into one positive category.

### Latent Markov Models and Their Application to Evaluating Diagnostics for *S. mansoni* Infection

LMM consists of a *structural model* for the latent infection states (analogous to the latent classes in Latent Class Analysis) and a *measurement model* for the observed indicators (these are the four diagnostic tools: the average of two KK measurements on three consecutive days and the CCA), conditional on latent infection state.

Let ***Y*** = (*y_i1t_,…, y_iPt_*) be a response pattern for the *i* ^th^ individual at time *t* on *P* observed binary indicators (in this study *P* = 4 binary diagnostic tests) with values ‘*0*’ and ‘*1*’ indicating negative and positive diagnostic test results, respectively.

We assume that for each individual the true underlying infection state, at each discrete time point *t* (where *t* = 1,…*T* and *T* = 3: baseline, nine weeks and two years) is explained by a latent categorical variable denoted by *C* with two latent infection states (i.e. those with *S. mansoni* infection and those without).

The responses to the (P×T) *y* indicators are assumed to be independent conditional on the latent infection state membership which in our analysis implies that the results from the four diagnostics are assumed to be independent conditional on the true underlying infection state both within and across time points. In addition, the latent categorical variable *C_i,t_* depends on *C_i,t-1_* but not on earlier latent categorical variables, known as the *first-order Markov* property. Under these assumptions, the probability of observing a particular response pattern **Y** for a randomly selected individual *i* is:
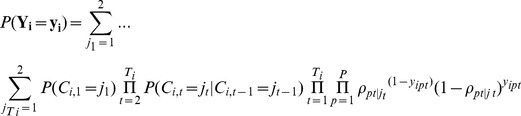
where 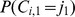 represents the baseline infection state prevalence at the first time point (i.e. baseline), and 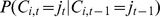 represents the transition probability to latent infection state *j_t_* at time *t* conditional on membership in latent infection state *j_t-1_* at time *t*-1. 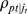 represents the diagnostic specificity for infection when the probability of the *p* diagnostic test is negative conditioned on *C_i,t_* representing the ‘Not Infected’ latent state. Similarly, the diagnostic sensitivity for infection of each of the four diagnostics is obtained when the probability of the *p* diagnostic test is positive conditioned on *C_i,t_* representing the ‘Infected’ latent state.

**Figure 1:**
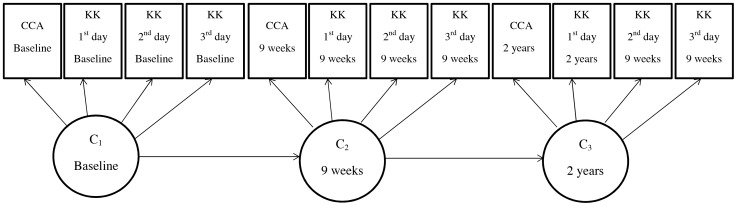
Latent Markov modelling path diagram. The variables in boxes represent the four observed categorical indicators of the latent categorical variables *C* at each time point *t*. The three arrows between the circled variables indicate the regression model for the latent categorical variable at time point *t* on the latent categorical variable at time point *t*-1.

Figure 1 presents our LMM in a path diagram.

### Computation

We fitted several LMMs using MPLUS v. 6.1 (Muthén & Muthén, Los Angeles) [31] with full information maximum likelihood in which we assumed that missing data were missing at random and making maximum use of data from individuals with incomplete data at the time points under study, for two different age groups: a) n = 167 children of age 7–16 years old and b) n = 273 adolescents and adults of age 17–76 years old. It is well known that different contact patterns with infected water bodies and consequently acquired exposure, immunity and susceptibility to infection might be experienced by different age groups [32].

We thus estimated the following sets of parameters for these two age groups:

1. Probabilities of positive or negative responses from KK and CCA diagnostic tests conditional on the latent ‘infection’ state at each time point (i.e. diagnostic sensitivities and specificities); in latent variable modelling literature these are called *item response probabilities*.
2. Transition probabilities of the latent infection states between the study time points i.e. from baseline to nine weeks and from nine weeks to two years.
3. The ‘true’ prevalence of *S. mansoni* infection at each time point.

### Model Selection and Classification of a “Trace” CCA Test Line

We selected the LMMs that optimally combined goodness of fit and parsimony as measured using the Akaike Information Criterion (AIC) and the Bayesian Information Criterion (BIC). [33] Both AIC and BIC are penalized-likelihood criteria. AIC is an estimate of a constant plus the relative distance between the unknown true likelihood function of the data and the fitted likelihood function of the model, so that a lower AIC means a model is considered to be better. BIC is an estimate of a function of the posterior probability of a model being true, under a particular Bayesian framework, so that a lower BIC means that a model is considered to be better. BIC penalizes model complexity more heavily and thus, whenever there is an inconsistency AIC indicates a preference for a more complex model than BIC [34]. Both criteria are based on assumptions and asymptotic approximations. Each, despite its heuristic usefulness, has therefore been criticized as having questionable validity for real-world data [34], [35]. In the current study, we examined AIC and BIC as well sample-size-adjusted BIC among models that were considered biologically plausible for the epidemiological settings under study (given the effect of one and two PZQ doses on schistosomiasis cure rates within the studied age groups).

**Figure 2:**
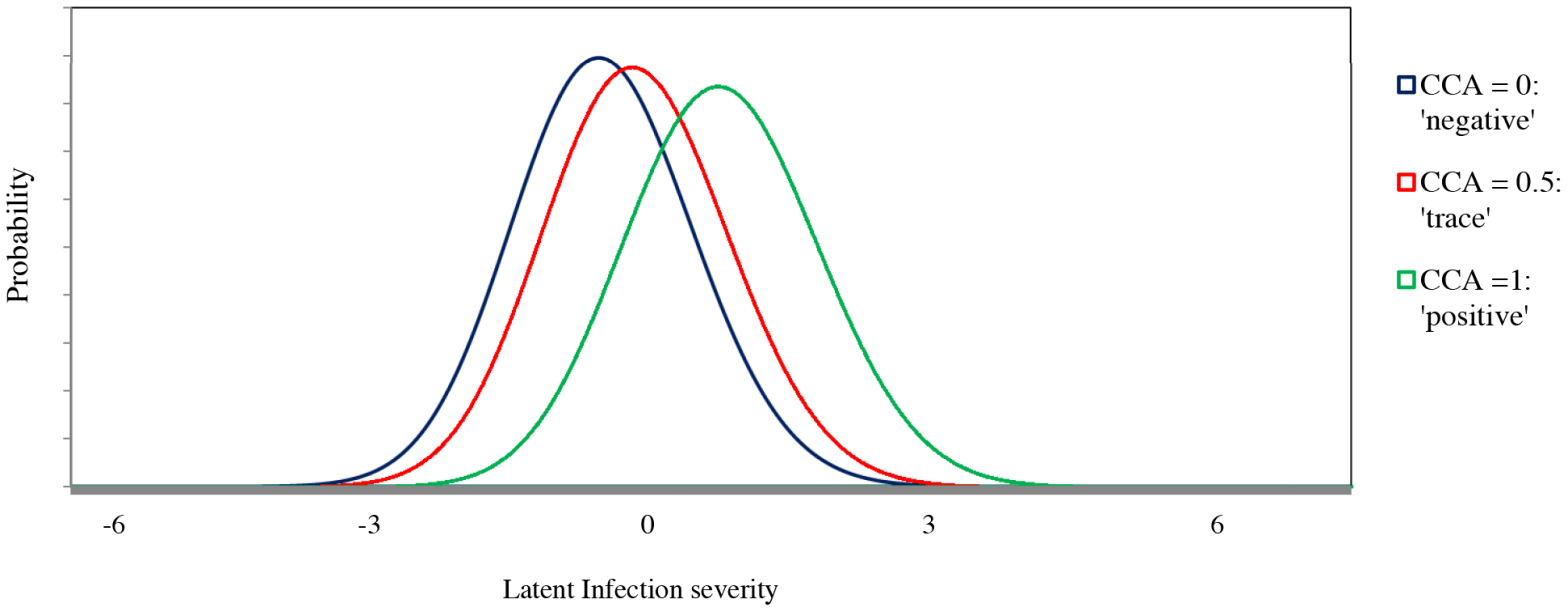
Posterior distributions of latent variables conditioned on the responses of the CCA test for children (n = 167). The latent variable represents a continuum of the infection severity characterized from the left to the right as to low or no infection up to high infection. The obtained scores in the horizontal axis are linked to ranking and not necessarily to the absolute displayed values.

**Figure 3:**
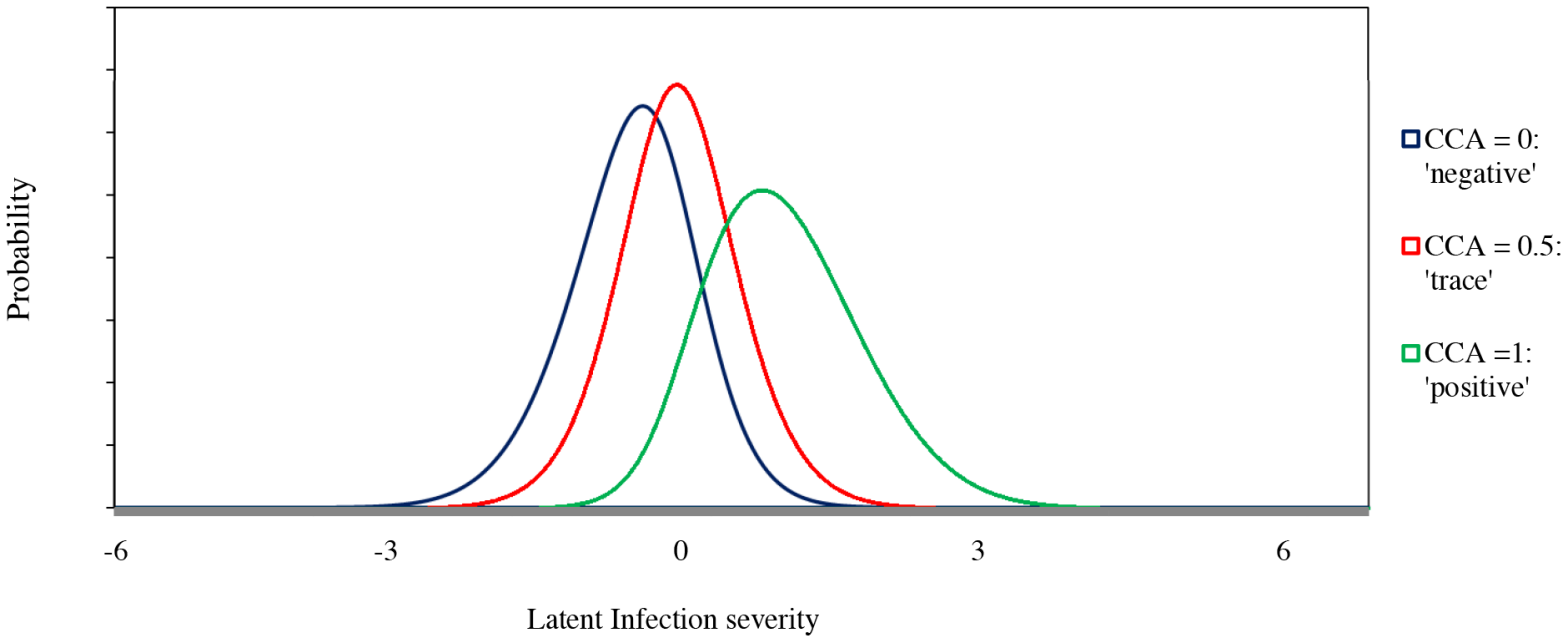
Posterior distributions of latent variables conditioned on the responses of the CCA test for adolescents and adults (n = 273). The latent variable represents a continuum of the infection severity characterized from the left to the right as to low or no infection up to high infection. The obtained scores in the horizontal axis are linked to ranking and not necessarily to the absolute displayed values.

We first tested whether the interpretation of a ‘trace’ CCA test line should be classified as positive or negative as several studies have reported on the ambiguity of infection status among those classified as ‘trace’ [16], [17], [36], [37]. For this problem we used a one factor analysis model [38] on the baseline data, to explore the interrelationships among the four diagnostic tests for both age groups, treating the three results of CCA (‘negative’, ‘trace’ and ‘positive’) as nominal (unordered). We plotted the posterior distribution of the latent variable given the three possible responses to CCA for children (Figure 2) and adolescents and adults (Figure 3), after estimating the one factor analysis model. Having observed the similarities in the posterior distributions for the ‘trace’ and ‘negative’ categories in Figures 2 and 3, we decided to treat ‘negative’ and ‘trace’ CCA as a single ‘negative’ category for the rest of this analysis. Similar statistical analysis might be useful for data related to point of care-CCA in order to further validate results and proceed with generalization of recommendations about trace results for the CCA in the field.

We subsequently fitted LMMs where we initially tested whether the item response probabilities (*ρ*'s, i.e. the sensitivities and specificities) assumed to be the same at baseline and two years but allowed to vary at nine weeks for both KK and CCA tests. In other words we tested the hypothesis of measurement invariance which assumes the equality of the parameters of the measurement model i.e. the conditional item response probabilities for the latent infection states at the different time points. We did not test for different *ρ*'s at each of the three time points (baseline, nine weeks and two years) because if they did differ at each time point the meaning of the latent infection states would have changed over time making the transition probabilities *τ*'s uninterpretable. The reason for this is that along with interpreting quantitative change over time in latent infection state membership (i.e. through the *τ*'s), it also becomes necessary to interpret change over time in the meaning of the latent infection state (i.e. which latent infection state represents the ‘true’ negatives and ‘true’ positives at each time point).

We also tested whether the provision of one or two PZQ doses affected the transition probabilities *τ*'s in the non-treatment intervals (nine weeks and two years) or only in the treatment interval (baseline to nine weeks) since there might be long-term benefits of two PZQ doses in the absence of snail control as in this community. Equal transition probability *τ*'*s* across each of the treatment intervals under study was not tested because this was not consistent with prior knowledge of the MDA impacts.

Although there was information identifying the household of each individual in the study, there were not enough data within each of the age groups studied to take into account between-household variability in the considered models.

## Results

The observed numbers of “true” and “false positives” and “true” and “false negatives”, and corresponding observed sensitivity and specificity, for the CCA diagnostic test are presented assuming, for illustration, the combination of three duplicate KK measures over the three consecutive days as being both 100% sensitive and 100% specific (Table 1).

When the sensitivities and specificities of single-day double KK results were estimated by fitting LMMs, the information criteria indicated that, for both age groups, the transition probability matrices [for the baseline-to-nine weeks follow-up (treatment interval) and for the nine weeks-to-two years follow-up (non-treatment interval)] depended on the provision of one or two PZQ doses. Similarly, the measurement invariance hypothesis for CCA was accepted based on the information criteria; however, the measurement invariance hypothesis was rejected for KK (i.e. the diagnostic performance of KK was found to vary over time but that of CCA was not). For further details on final model selection, see Table S1. The estimated sensitivities and specificities for CCA and KK tests, from the best LMM for each age group are presented in Table 2. Based on these estimates and if one took the crude average of the estimated sensitivities and specificities of the two diagnostic measures and time points under examination, CCA overall was found to be more sensitive but less specific than double KK from a single faecal sample for both age groups. The sensitivity of double KK from a single faecal sample was found to be lower at nine weeks than at baseline and two years for both age groups in all three days for adolescents and adults and for two of the three days for children.

The estimated transition probabilities between the latent infection states over time are presented in Table 3. Diagonal elements represent the probabilities of remaining in the same latent infection state at time *t* as at the previous time (*t*-1). The majority of adolescents and adults apparently remained, over the non-treatment interval, in the same latent infection state. Among those infected at baseline in both age groups, two PZQ doses produced a higher probability of clearance of infection at nine weeks than just one PZQ dose; for children these probabilities were 0.717 (2 doses) and 0.458 (1 dose) (see Table 3A), while for adolescents and adults these probabilities were 0.846 (2 doses) and 0.561 (1 dose) (see Table 3B).

**Figure 4:**
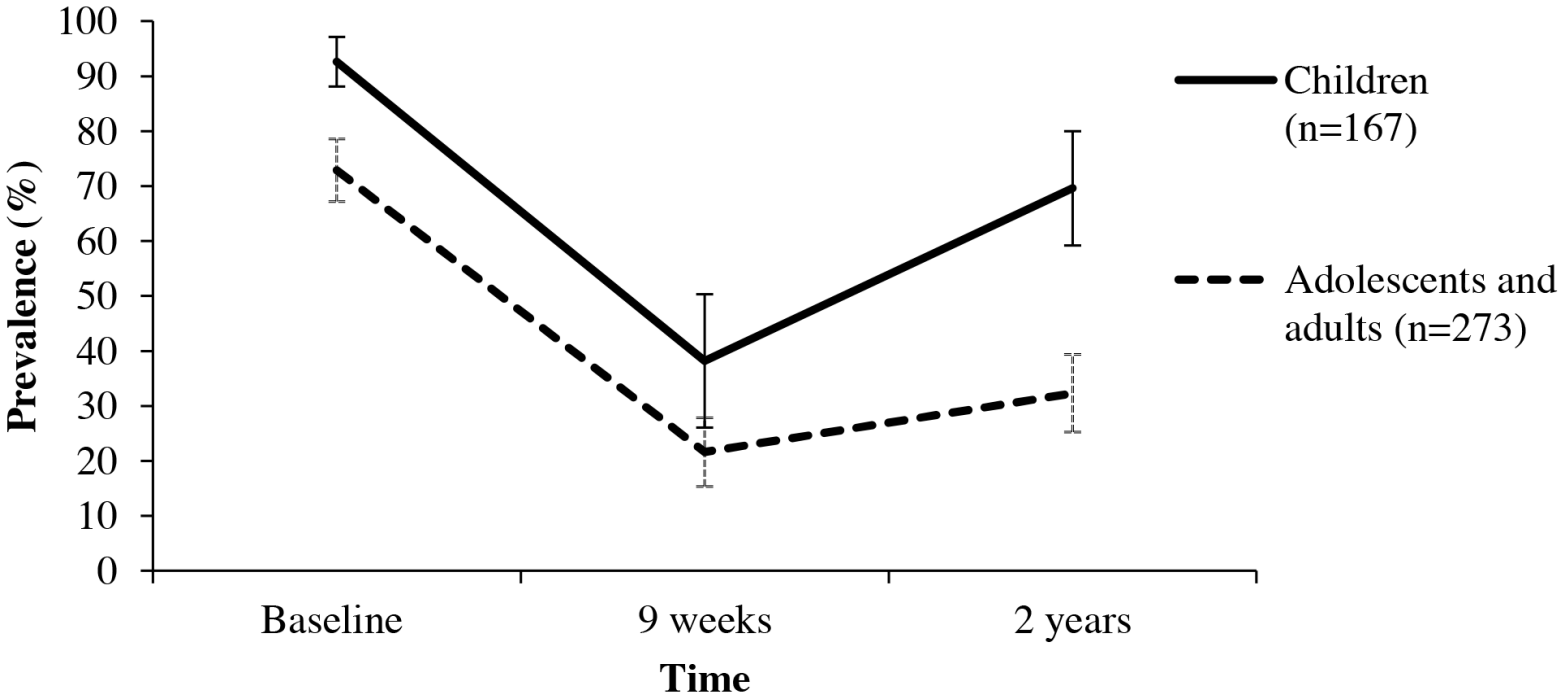
LMM estimated “true” S. mansoni prevalence for both age groups (ages 7–16 and 17–76) with 95% confidence intervals over time.

The *S. mansoni* prevalence estimate for each time point and each age group is presented in Figure 4 with 95% confidence intervals. Following treatment the prevalence of *S. mansoni* infection decreased dramatically between baseline to nine weeks for both age groups. Among children there is a substantial rebound by two years. These patterns are also reflected in the transition probabilities in Table 3.

Although visual examination of Table 4 superficially suggests that the LMM estimate conflicts with that obtained based on the 6 KK measurements (assumed positive if one or more of the six measurements was positive), further calculations are required to underpin full interpretation of the results.

**Table 4:** Estimated *S. mansoni* prevalences based on 6 KK measurements and based on the LMM.

Source of the estimate	Baseline	9 weeks	2 years
	***Children***		
*6 KK measurements*	97.6	62.1	90.8
*Latent Markov model*	92.6	38.0	67.9
	***Adolescents and Adults***		
*6 KK measurements*	83.9	30.0	56.5
*Latent Markov model*	72.9	22.5	31.3

For instance, for children at *baseline*, the estimated prevalence based on the LMM is 92.6%.

One would expect the following percentage of **true** positives:
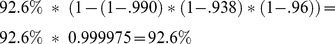
where 0.990, 0.938 and 0.960 are the estimated sensitivities of two KK measurements from days one, two and three, respectively

One would expect the following percentage of **false** positives:
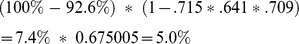
where 0.751, 0.641 and 0.709 are the estimated specificities of two KK measurements from days one, two and three, respectively.

Thus, based on the estimated sensitivities, specificities and the assumption that results from days one, two and three, were independent, conditional on the true status, the LMM predicts that the estimated prevalence based on 6 KK measurements would be **97.6%** (92.6% true positive and 5.0% false positives). This is highly consistent with the estimate obtained from the 6 KK measurements: **97.6%.**

For the remaining time points and age groups please see in Supporting information.

## Discussion

This study analysed a longitudinal detailed dataset from Uganda in which one or two doses of PZQ treatment were provided at baseline using LMM that accounted for the longitudinal study design and the measurement error in the diagnostic methods under study. Our primary objective was to assess the CCA diagnostic accuracy at baseline and at two follow-up times after treatment but we also evaluate double KK faecal slides over three consecutive days for *S. mansoni* infection. To our knowledge, this is the first study which provides rigorous model-based diagnostic performance of CCA and single-day double KK measurements over three consecutive days for the diagnosis of *S. mansoni* infection in two different age groups pre- and post- PZQ treatment.

CCA's diagnostic performance was found to be constant over time and overall approximately 90% sensitive but less specific than single-day double KK faecal slides for *S. mansoni* infection in both age groups. Day-to-day variation in faecal egg output among *Schistosoma* parasites [7]–[9] has been shown to be greater [11] and with lower sensitivity of KK after PZQ treatment [8]. The single-day double KK sensitivity is likely to depend strongly on the observed prevalence [8], [15], [39]. Our study confirmed these findings and arguments and showed clearly that sensitivity of single-day double KK was much lower at nine weeks than at baseline and two years for both age groups in all three days for adolescents and adults and for two of the three days for children while its specificity increased after PZQ treatment (Table 2).

These findings bridge existing gaps in schistosomiasis diagnostics research such as for instance the lack of CCA evaluation in adolescents and adults and the lack for evidence for its capacity to determine if a person has been cured after treatment, as previously highlighted [4], [17]. The current analysis provides model-based estimates of sensitivity and specificity and their uncertainties (through the provision of 95% confidence intervals) without assuming any gold standard diagnostic test in the statistical analysis. The exact numbers of false positive and false negative results are almost always unknown and thus in the current study we estimated rather than assumed values for the parameters displayed in Tables 2 and 3 (sensitivities, specificities and transition probabilities) [40], [41]. Without quantification of the uncertainties regarding the performance of the key diagnostic tests, generalization of epidemiological results and development of useful recommendations for which diagnostics to use and at which stages of schistosomiasis control are hampered.

This approach evaluates the risk of potential misinterpretation with regards to diagnosis of *S. mansoni* infection through CCA or KK in this endemic setting pre- and post- PZQ treatment as the numbers and infection intensities are brought down [12]. For instance, results in Table 1 demonstrate that by using 6 KK measurements over three days as the gold standard (i.e. assuming 100% sensitivity and 100% specificity), the resulting empirical estimates of CCA sensitivity and specificity are mistakenly shown to vary over time. We do not expect that the clearance of the antigen is influenced by treatment. Furthermore, the hypothesis of measurement invariance was not rejected based on information criteria for the fitted LMMs (see Text S1 and Table S1 in Supporting Information).

Glinz *et al.* 2010 [42] discussed possible reasons for false positive diagnoses from KK tests. Results from model in Table 2 clearly indicate that the specificity of single-day double KK measurements is lower than 100%. This means that despite highly qualified and skilled co-workers, contamination of stool sieves in the field and data entry errors cannot completely be avoided. Because our estimated specificities of single-day double KK measurements were less than 100%, the estimated ‘true’ *S. mansoni* prevalence (Figure 4) is lower at each time points than the estimated prevalence obtained assuming that any individual with positive results on one or more of the six KK tests conducted over three consecutive days was infected (Tables 1 and 4 ). This is in accordance with work on diagnostic performance of KK for animal schistosomiasis infection [14]. Previous work using stochastic models have demonstrated that the sensitivity of the KK would vary according to the number of stool samples provided [7]–[9] and the ‘true’ *S. mansoni* prevalence at baseline can be calculated using the De Vlas pocket chart [8]. For the children group, the chart is not applicable since the observed prevalence in this study is beyond the limits where the De Vlas model is valid and thus we cannot compare it with the estimates of ‘true’ prevalence from our model (Figure 4). For the adolescents and adults group however estimates of the ‘true’ prevalence (Figure 4) were not consistent with this chart because it was based on an assumption of 100% KK specificity.

The estimated transition probability matrices (Table 3) provide parsimonious yet important insights into the re-infection and cure rates in the two age groups. The cure rate was higher in adolescents and adults than in children following treatment. This can be explained by the fact that those infected in the older age group had lower burdens than the infected children and would be therefore more likely to become negative. From an immunological perspective view, it can be argued that the older age group are more likely to have developed protective immune response and are therefore more efficient in affecting and killing the worms [43], [44]. The quantification of the additional benefit of a second PZQ dose in reducing infection levels for both age groups was demonstrated by higher transition probabilities from infected to non-infected among those who received two PZQ doses compared to those who received one PZQ dose within the treatment interval. For the non treatment interval (i.e. between nine weeks and two years) there were no differences in reinfection or cure rates among those who received one or two PZQ doses. As the LMMs are estimated using an iterative algorithm, at each step of the algorithm, estimated values very close to 0 or 1 can create estimation instability and therefore are automatically from MPLUS fixed to 0 and 1 respectively in order to avoid non-convergence of the estimation algorithm. Consequently, such values should be treated with caution due to computational limitations in these categories during the model estimation. Finally, we recognize that the results of this study depend upon the assumption of conditional independence assumed by the models fitted here. Once one conditions on the latent infection state, we believe though that there are good reasons to assume that KK and CCA are independent as *Schistosoma* eggs and antigens are excreted through different routes in the human body, for instance.

To conclude, in the absence of a diagnostic gold standard this study has demonstrated that LMMs can be useful for the evaluation of available diagnostic tools for *S. mansoni* infection. More generally, we recommend LMMs to be used for the evaluation of diagnostic tests of other diseases without gold standard diagnostic tools whenever longitudinal data are available as such modelling permits questions about changes in true infection states and test the measurement invariance hypothesis of the diagnostic tests of interest over time - making them very useful tools indeed for control program M&E research [21]. Further work in evaluating the trace result and the ability of CCA to quantify intensity of infection is also warranted in the M&E of schistosomiasis control programs and dynamic latent factor models (such models would assume continuous hypothetical constructs or typologies-i.e. intensity of infection) might be appropriate statistical methods for the analysis of relevant data. Similar studies should be considered at other sites in order to build on our results. We found that the CCA diagnostic performance remained constant after provision of PZQ treatment and that the test is overall more sensitive but less specific than single-day double KK for the diagnosis of *S. mansoni* infection. In line with the results from our study and those of a recent multi-country cross-sectional study which showed that for lower *S. mansoni* intensity settings the CCA sensitivity was demonstrated to be higher than KK [17], we recommend that CCA to be used for mapping surveys of *S. mansoni* infection. As public health measures are aimed at the elimination of residual foci of schistosomes, data generated using diagnostics with high specificity will be required to avoid unnecessarily prolonging MDA and wasting scarce resources [5]. Detection of parasite-specific DNA [45], [46] or circulating anodic antigen in serum or urine [47] might present alternative opportunities in schistosomiasis elimination programs and further evaluations of these diagnostics merit attention.

## Supporting Information

Table S1 Different fitted LMMs.
(DOCX)

Text S1 Comparison of Alternative Latent Structures of LMMs.
(DOCX)

Text S2 Validation of LMM estimates with observed 6 KK measurements.
(DOCX)

Text S3 Comparisons of estimated *S. mansoni* prevalences with the De Vlas pocket chart.
(DOCX)
