# Supplementary material for: A Latent Markov Modelling Approach to the Evaluation of Circulating Cathodic Antigen Strips for Schistosomiasis Diagnosis Pre- and Post-Praziquantel Treatment in Uganda
Source: PLoS Comput Biol. 2013 Dec 19;9(12):e1003402. doi: 10.1371/journal.pcbi.1003402 (PMC3868541; doi:10.1371/journal.pcbi.1003402)
Supplement: Table S1 — Different fitted LMMs. (DOCX) [file pcbi.1003402.s001.docx]

**TABLE S1.**

|  | Children (N=167) | | | | | |
| --- | --- | --- | --- | --- | --- | --- |
| *Model* | *Transition matrices* | *r* | *LL* | *BIC* | *AIC* | *Sample adjusted BIC* |
| *1** | 4 | 16 | -851.337 | **1784.561** | 1734.673 | 1733.903 |
| *2†* | 4 | 22 | -842.013 | 1796.623 | 1728.027 | 1726.967 |
| *3‡* | 4 | 25 | -838.10 | 1805.571 | **1727.621** | **1726.417** |
| *4•* | 4 | 18 | -846.914 | 1785.952 | 1729.828 | 1728.962 |
| *5↕* | 3 | 14 | -857.072 | 1785.795 | 1742.143 | 1741.469 |
| *6⌂* | 3 | 20 | -849.499 | 1801.358 | 1738.998 | 1738.035 |
| *7⌡* | 3 | 16 | -852.977 | 1787.843 | 1737.955 | 1737.184 |

|  | Adolescents & Adults (N= 273) | | | | | |
| --- | --- | --- | --- | --- | --- | --- |
| *Model* | *Transition matrices* | *r* | *LL* | *BIC* | *AIC* | *Sample adjusted BIC* |
| *1** | 4 | 16 | -1506.066 | 3101.884 | 3044.133 | 3051.152 |
| *2†* | 4 | 22 | -1460.279 | **3043.966** | **2964.558** | **2974.210** |
| *3‡* | 4 | 25 | -1459.448 | 3059.133 | 2968.897 | 2979.864 |
| *4•* | 4 | 18 | -1504.943 | 3110.857 | 3045.886 | 3053.783 |
| *5↕* | 3 | 14 | -1529.243 | 3137.018 | 3086.486 | 3092.628 |
| *6⌂* | 3 | 20 | -1492.209 | 3096.608 | 3024.419 | 3033.193 |
| *7⌡* | 3 | 16 | -1528.763 | 3147.278 | 3089.527 | 3096.546 |

Abbreviations: *r,* number of free parameters; *LL*, corresponding maximum log-likelihood; *BIC,* Bayesian Information Criterion; *AIC,* Akaike Information Criterion

**Model 1:* full measurement invariance i.e. all item response probabilities (i.e. for all diagnostic tests) are constant over time. In addition transition probabilities for both treatment and non treatment intervals, depend on the number of treatments;

*†Model 2:* partial measurement invariance i.e. KK response probabilities vary at 9 weeks but are constant at baseline and 2 years while CCA response probabilities are constant during the 3 time points of interest (i.e. baseline, 9 weeks and 2 years). In addition transition probabilities for both treatment and non treatment intervals, depend on the number of treatments;

*‡Model 3:* Same as Model 2 with the additional scenario that the ‘true’ prevalence of *S. mansoni* infection at each time point depend on the number of treatments; these effects (i.e. whether the ‘true’ prevalence of *S. mansoni* infection at each time point depended on the number of treatments) were not significant.

*•Model 4:* partial measurement invariance i.e. CCA response probabilities vary at 9 weeks but are constant at baseline and 2 years while KK response probabilities are constant during the 3 time points of interest. In addition transition probabilities for both treatment and non treatment intervals, depend on the number of treatments;

*↕Model 5:* full measurement invariance i.e. all item response probabilities (i.e. for all diagnostic tests) are constant over time. In addition, from 9 weeks to 2 years, transition probabilities do not depend on number of treatments;

*⌂Model 6:* partial measurement invariance i.e. KK response probabilities vary at 9 weeks but are constant at baseline and 2 years while CCA response probabilities are constant during the 3 time points of interest (i.e. baseline, 9 weeks and 2 years). In addition, from 9 weeks to 2 years transition probabilities do not depend on number of treatments;

*⌡Model 7:* partial measurement invariance i.e. CCA response probabilities vary at 9 weeks but are constant at baseline and 2 years while KK response probabilities are constant during the 3 time points of interest. In addition, from 9 weeks to 2 years transition probabilities do not depend on number of treatments.
