# Supplementary material for: A Latent Markov Modelling Approach to the Evaluation of Circulating Cathodic Antigen Strips for Schistosomiasis Diagnosis Pre- and Post-Praziquantel Treatment in Uganda
Source: PLoS Comput Biol. 2013 Dec 19;9(12):e1003402. doi: 10.1371/journal.pcbi.1003402 (PMC3868541; doi:10.1371/journal.pcbi.1003402)
Supplement: Text S1 — Comparison of Alternative Latent Structures of LMMs. (DOCX) [file pcbi.1003402.s002.docx]

**Text S1**

Tables below report the number of transition matrices, the LL, the BIC, AIC and sample adjusted BIC value for the LMMs that were obtained during the model selection. Highlighted values in bold indicate the lowest obtained information criteria. The *r* is calculated by P_η_: number of latent infection state prevalences; P_ρ_ number of item response probabilities and P_τ_ number of transition probabilities estimated.

The formula for the sample adjusted BIC value is nearly identical to the formula for BIC with the difference that it replaces in the latter *n* with *n** (where n* = (n + 2) / 24).

Based on these information criteria and number of parameters involved for both examined age groups, we finally selected Model 2 for results interpretation. Although for children, AIC and sample adjusted BIC were slightly smaller for Model 3 if compared to Model 2, the use of 3 more additional parameters does not significantly improve the model fit (p-value from likelihood ratio test for children was 0.050 while for adolescents and adults this was 0.645).

In addition, for the final presented models in both age groups we tested the absolute model fit as indicated by the Likelihood Ratio Chi-Square and the p-value was equal to 1.000 which indicates not to reject the null hypothesis that this is the population model that produced the observed data.

Furthermore, before selecting and presenting Model 2 (i.e. model with partial measurement invariance-i.e. only KKs ρ’s varied at 9 weeks compared to baseline and 2 years) we also compared the obtained transition probabilities from both Model 2 and Model 1 When comparing Models 1 and 2, the relevant estimated transition probabilities remained similar which gives us the confidence to assign a unique meaning to the latent infection states across time.

Finally, we believe that hypotheses represented in Model 2, are not biologically unreasonable. In fact we think that they are conceptually appealing since at baseline and 2 years the ‘true’ *S. mansoni* prevalence levels would not be that different and thus assuming measurement invariance between these 2 time points seems plausible.
