# Supplementary material for: A Latent Markov Modelling Approach to the Evaluation of Circulating Cathodic Antigen Strips for Schistosomiasis Diagnosis Pre- and Post-Praziquantel Treatment in Uganda
Source: PLoS Comput Biol. 2013 Dec 19;9(12):e1003402. doi: 10.1371/journal.pcbi.1003402 (PMC3868541; doi:10.1371/journal.pcbi.1003402)
Supplement: Text S2 — Validation of LMM estimates with observed 6 KK measurements. (DOCX) [file pcbi.1003402.s003.docx]

**Text S2**

For children at *9 weeks*, the estimated prevalence based on the LMM is 38.0%.

One would expect the following percentage of **true** positives:

38.0% * (1 – (1-.820)*(1-.941)*(1-.726)) = 38.0% * 0.99709 = 37.9%

where 0.820, 0.941 and 0.726 are the estimated sensitivities of two KK measurements from days 1, 2 and 3, respectively

One would expect the following percentage of **false** positives:

(100% - 38.0%) * (1 –.876*.904*.800) = 62.0% * 0.366477 = 22.7%

where 0.876, 0.904 and 0.800 are the estimated specificities of two KK measurements from days 1, 2 and 3, respectively.

Thus, based on the estimated sensitivities, specificities and the assumption that results from days 1, 2 and 3 were independent, conditional on the true status, the LMM predicts that the estimated prevalence based on 6 KK measurements would be **60.6%** (37.9% true positive and 22.7% false positives). This is highly consistent with the estimate obtained from the 6 KK measurements: **62.1%.**

For children at *2 years*, the estimated prevalence based on the LMM is 67.9%.

One would expect the following percentage of **true** positives:

67.9% * (1 - (1-.990)*(1-.938)*(1-.96)) = 67.9% * 0.999975 = 67.9%

where 0.990, 0.938 and 0.960 are the estimated sensitivities of two KK measurements from days 1, 2 and 3, respectively

One would expect the following percentage of **false** positives:

(100% - 67.9%) * (1 -.715*.641*.709) = 32.1% * 0.675005 = 21.7%

where 0.751, 0.641 and 0.709 are the estimated specificities of two KK measurements from days 1, 2 and 3, respectively.

Thus, based on the estimated sensitivities, specificities and the assumption that results from days 1, 2 and 3 were independent, conditional on the true status, the LMM predicts that the estimated prevalence based on 6 KK measurements would be **89.6%** (67.9% true positive and 21.7% false positives). This is highly consistent with the estimate obtained from the 6 KK measurements: **90.8%.**

Similarly for adolescents and adults at *baseline***,** the estimated prevalence based on the LMM is 72.9%.

One would expect the following percentage of **true** positives:

72.9% * (1 - (1-.940)*(1-.927)*(1-.954)) = 72.9% * 0.999979 = 72.9%

where 0.940, 0.927 and 0.954 are the estimated sensitivities of two KK measurements from days 1, 2 and 3, respectively

One would expect the following percentage of **false** positives:

(100% - 72.9%) * (1 -.815*.858*.859) = 27.1% * 0.399327 = 10.8%

where 0.815, 0.858 and 0.859 are the estimated specificities of two KK measurements from days 1, 2 and 3, respectively.

Thus, based on the estimated sensitivities, specificities and the assumption that results from days 1, 2 and 3 were independent, conditional on the true status, the LMM predicts that the estimated prevalence based on 6 KK measurements would be **83.7.%** (72.9% true positive and 10.8% false positives). This is highly consistent with the estimate obtained from the 6 KK measurements: **83.9%.**

For adolescents and adults at *9 weeks*, the estimated prevalence based on the LMM is 22.5%.

One would expect the following percentage of **true** positives:

22.5% * (1 - (1-.497)*(1-.600)*(1-.541)) = 22.5% * 0.907649 = 20.4%

where 0.497, 0.600 and 0.541 are the estimated sensitivities of two KK measurements from days 1, 2 and 3, respectively

One would expect the following percentage of **false** positives:

(100% - 22.5%) * (1 -.956*.987*.957) = 77.5% * 0.097002 = 7.5%

where 0.956, 0.987 and 0.957 are the estimated specificities of two KK measurements from days 1, 2 and 3, respectively.

Thus, based on the estimated sensitivities, specificities and the assumption that results from days 1, 2 and 3 were independent, conditional on the true status, the LMM predicts that the estimated prevalence based on 6 KK measurements would be **27.9%** (20.4% true positive and 7.5% false positives). This is highly consistent with the estimate obtained from the 6 KK measurements: **30.0%.**

For adolescents and adults at *2 years*, the estimated prevalence based on the LMM is 31.3%.

One would expect the following percentage of **true** positives:

31.3% * (1 - (1-.940)*(1-.927)*(1-.954)) = 31.3% * 0.999799 = 31.3%

where 0.940, 0.927 and 0.954 are the estimated sensitivities of two KK measurements from days 1, 2 and 3, respectively

One would expect the following percentage of **false** positives:

(100% - 31.3%) * (1 -.815*.858*.859) = 68.7% * 0.399327 = 27.4%

where 0.815, 0.858 and 0.859 are the estimated specificities of two KK measurements from days 1, 2 and 3, respectively.

Thus, based on the estimated sensitivities, specificities and the assumption that results from days 1, 2 and 3 were independent, conditional on the true status, the LMM predicts that the estimated prevalence based on 6 KK measurements would be **58.7%** (31.3% true positive and 27.4% false positives). This is highly consistent with the estimate obtained from the 6 KK measurements: **56.5%.**
