# Supplementary material for: A Latent Markov Modelling Approach to the Evaluation of Circulating Cathodic Antigen Strips for Schistosomiasis Diagnosis Pre- and Post-Praziquantel Treatment in Uganda
Source: PLoS Comput Biol. 2013 Dec 19;9(12):e1003402. doi: 10.1371/journal.pcbi.1003402 (PMC3868541; doi:10.1371/journal.pcbi.1003402)
Supplement: Text S3 — Comparisons of estimated S. mansoni prevalences with the De Vlas pocket chart. (DOCX) [file pcbi.1003402.s004.docx]

**Text S3**

Finally, if one also compares the estimates of ‘true’ prevalence for the adolescents and adults using the De Vlas pocket chart ^1^ - where calculations were also based in an assumption of 100 % KK specificity- these are much closer to the 6 KK measurements (assumed positive if 1 or more of the 6 measurements was positive) at baseline and 2 years. We do not comment on the 9 weeks since the chart would not be valid after population chemotherapy, during a variable period that depends on the type of intervention and the level of re-infection. This chart is also not applicable on the children’s data in this study, since the observed prevalence is beyond the limits where the deVlas model is valid.

1. De Vlas SJ, Gryseels B. Underestimation of *Schistosoma mansoni* prevalences. *Parasitol Today* 1992;**8**(8):274-7
